# Transluminal forceps biopsy for a pancreaticojejunostomy stricture using a novel forceps biopsy device

**DOI:** 10.1055/a-2638-5838

**Published:** 2025-07-10

**Authors:** Takeshi Ogura, Kimi Bessho, Nobuhiro Hattori, Yuki Uba, Hiroki Nishikawa

**Affiliations:** 1 38588 Endoscopy Center, Osaka Medical and Pharmaceutical University Hospital, Takatsuki, Japan; 2 13010 2nd Department of Internal Medicine, Osaka Medical and Pharmaceutical University, Takatsuki, Japan

Endoscopic ultrasound (EUS)-guided pancreatic duct drainage (EUS-PDD) is indicated in cases of pancreatic duct obstruction, such as a pancreaticojejunostomy stricture, where endoscopic retrograde cholangiopancreatography (ERCP) has failed 1 2 3 4 5 . During the EUS-PDD procedure, leakage of pancreatic juice can occur until the stent has been deployed. Various procedures, such as stone removal or forceps biopsy, may increase the risk of adverse events. Transluminal biopsy may also be challenging, because matching the axes of the stricture site and the biopsy device is sometimes difficult, and there is a risk of puncturing the drainage tract. To overcome this, a novel forceps biopsy device has become available in Japan (ERCP Guide Sheath; Umidas, Kanagawa, Japan) ( Fig. 1 ). The diameter of the outer sheath of this device is 8.5 Fr, and the inner sheath is 6.0 Fr. In addition, the tip of this device is extremely tapered, conforming to a 0.035-inch guidewire. These characteristics allow penetration of stricture sites, and after removal of the inner sheath, various devices can be inserted. Side holes are also provided for the injection of contrast medium. This device can prevent pancreatic juice leakage and allow transluminal biopsy to be performed. Here, we describe transluminal biopsy in a pancreaticojejunostomy stricture.

**Fig. 1:**
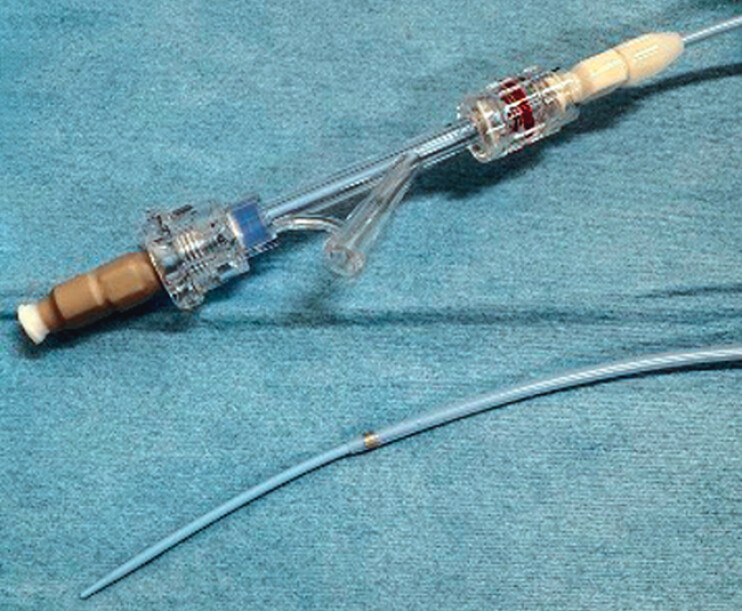
The novel forceps biopsy device (ERCP Guide Sheath, Umidas, Kanagawa, Japan). The diameter of the outer sheath of this device is 8.5 Fr, and that of the inner sheath is 6.0 Fr. In addition, the tip of this device is extremely tapered, conforming to a 0.035-inch guidewire.

A 49-year-old man was admitted to our hospital due to pancreatitis caused by a pancreaticojejunostomy stricture. He had undergone pancreaticoduodenectomy to treat intraductal papillary mucinous carcinoma (IPMC) 10 months earlier. EUS-PDD was performed. After 2 weeks, to differentiate between recurrence of IPMC and benign stricture, transluminal biopsy was attempted. First, a 0.025-inch guidewire was deployed beside the EUS-PDD stent using an ERCP catheter. Then, the EUS-PDD stent was removed using a forceps biopsy device, and an ERCP guide sheath was inserted into the main pancreatic duct ( Fig. 2 ). The inner sheath was then removed, and it was possible to insert the forceps biopsy device smoothly into the main pancreatic duct through the ERCP guide sheath ( Fig. 3 ). To identify the stricture site, contrast medium was injected through the outer sheath ( Fig. 4 ). Transluminal forceps biopsy was then successfully performed ( Fig. 5 ), followed, finally, by stent deployment. The stricture was diagnosed histologically as a benign stricture ( Video 1 ).

**Fig. 2:**
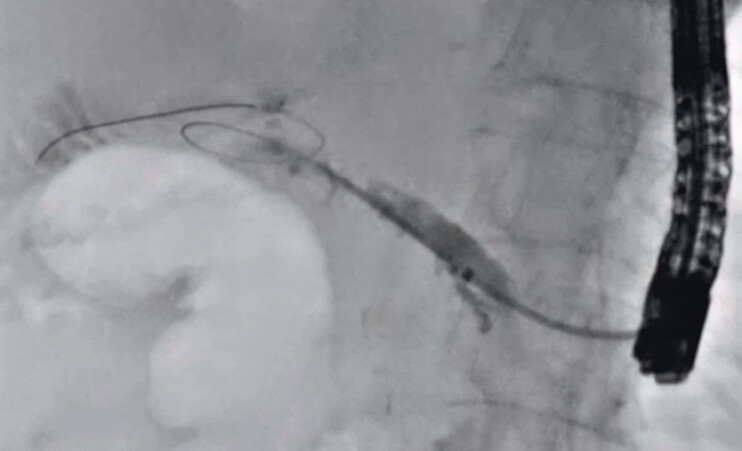
The ERCP guide sheath is inserted into the main pancreatic duct.

**Fig. 3:**
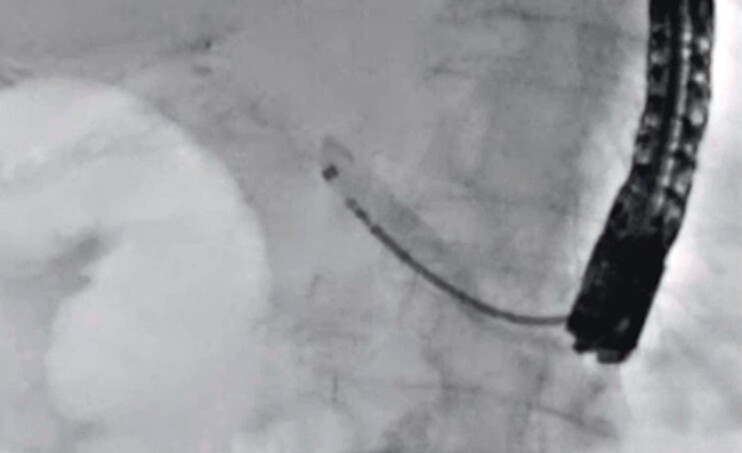
The forceps biopsy device can be inserted smoothly into the main pancreatic duct through the ERCP guide sheath.

**Fig. 4:**
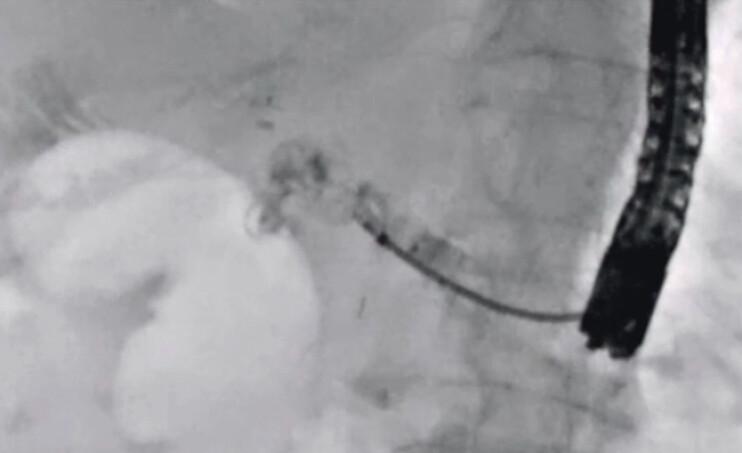
To identify the stricture site, contrast medium is injected through the outer sheath.

**Fig. 5:**
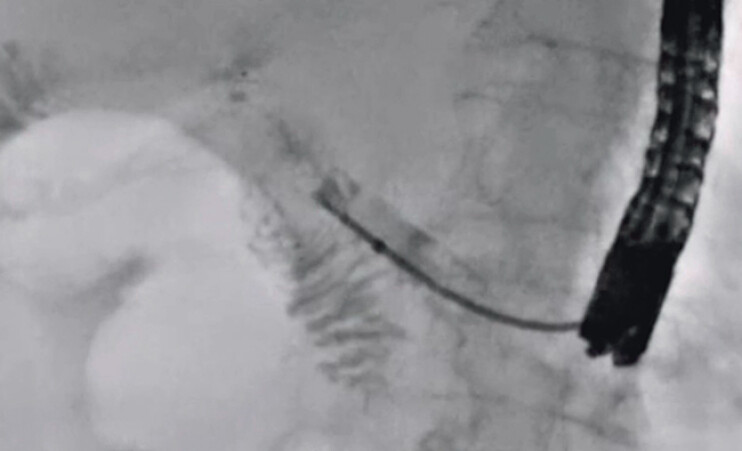
Transluminal forceps biopsy is successfully performed.

Transluminal forceps biopsy for a pancreaticojejunostomy stricture using a novel forceps biopsy device.Video 1

In conclusion, an ERCP guide sheath may be useful for forceps biopsy not only under ERCP, but also when using a transluminal approach.

Endoscopy_UCTN_Code_TTT_1AR_2AD

## Notes

Endoscopy E-Videos https://eref.thieme.de/e-videos
*E-Videos* is an open access online section of the journal *Endoscopy* , reporting on interesting cases and new techniques in gastroenterological endoscopy. All papers include a high-quality video and are published with a Creative Commons CC-BY license. Endoscopy E-Videos qualify for HINARI discounts and waivers and eligibility is automatically checked during the submission process. We grant 100% waivers to articles whose corresponding authors are based in Group A countries and 50% waivers to those who are based in Group B countries as classified by Research4Life (see: https://www.research4life.org/access/eligibility/ ). This section has its own submission website at https://mc.manuscriptcentral.com/e-videos .
